# Time after time, the best–worst scaling method is more reliable than ranking

**DOI:** 10.3758/s13428-026-03167-x

**Published:** 2026-09-01

**Authors:** Garston Liang, Mackenzie Glover, Guy E. Hawkins

**Affiliations:** 1 Discipline of Psychology, University of Technology Sydney, Building 20, 100 Broadway, Ultimo, New South Wales 2008 Australia; 2 Discipline of Psychology, University of Newcastle, Callaghan, Australia

**Keywords:** Ranking, Best–worst scaling, Preference elicitation, Reliability

## Abstract

Two popular methods of preference elicitation are rankings and best–worst scaling (BWS). Rankings, while simple and widely adopted, can be burdensome with larger item sets and fail to capture indifference between options that are neither loved nor hated. Best–worst scaling is a survey method that sidesteps the set size problem by capitalizing on people’s natural capacity to identify preferences at the extremes. Across three experiments, our primary finding is that elicited preferences for *ranking* and *BWS* methods align, and that BWS methods provide additional resolution to resolve the indifference between middling options where rankings can struggle as well as the relative importance of each option. Moreover, we show that BWS methods exhibit greater test–retest reliability compared to rankings, even over time frames as short as minutes. Taken together, our results privilege BWS as a reliable and readily accessible alternative to ranking methods for preference elicitation.

## Introduction

If Christmas dinner conversation ever needed more spice, arguing over the best era of music might just do it. All of a sudden, an ordinarily quiet uncle rambles on about The Beatles, music of someone’s good old days is clearly the best, and modern pop music is unquestionably the worst. This might not be the most agreeable conversation between generations (Van Dam, 2024) but, perhaps, it is a familiar one where the range of preferences is immediately clear and how one asks about these preferences matters. Beyond the dinner table, eliciting preferences is a foundational component of understanding a range of complex and more consequential decisions, including health care choices, consumer preferences, and political polling (Cooper & Hawkins, 2019; Ryan, 2004; Viney, Lancsar, & Louviere, 2002).

Preference elicitation has a rich history in the psychological and behavioral economics literature (Edwards, 1954; Von Neumann & Morgenstern, 2007). A common theoretical framework assumes that individuals have stable internal representations of their preferences. These preferences are *revealed* through behavioral measurement, where variability arises from the particular measurement tool. Within this framework, the elicitation method provides a window into people’s underlying representations, albeit with varying degrees of opacity.

In direct contrast, the *constructed preferences* view holds that while preferences can be obtained, they are constructed in the instant of measurement and so packaged with the conditions of the moment. Unlike the first perspective, constructed preferences need not be coherent over time as environmental features of that decision moment such as the available choice options, particular framing of the options, or the respondent themselves directly influences the elicited preferences (Lichtenstein & Slovic, 1971; McKenzie, Sher, Leong, & Müller-Trede, 2018; Slovic, 1995).

Both theoretical perspectives agree that the choice of elicitation method is centrally important, but given the many methods for eliciting preferences, a critical question is: to what extent do common methods agree with one another?

In this paper, our primary interest is test–retest reliability. We compare two popular methods of preference elicitation, namely *ranking* and *best–worst scaling*. Ranking tasks are ubiquitous and favored for their relative simplicity (Lee, Steyvers, De Young, & Miller, 2012; Montgomery, Bradford, & Lee, 2024). In everyday life, you might rank your favorite cafes, your favorite season of a television show, or the best movies of the year (Selker, Lee, & Iyer, 2017). Hiring panels rank preferred candidates and in the Australian preferential voting system, the House of Representatives are decided by citizens ranking a list of candidates from most to least preferred. For only a handful of options, e.g., one’s top three cafes, rankings can be easily obtained from relatively few comparisons. However, rankings can be muddied by indifference between options and, particularly for larger sets, the number of comparisons drastically increases, e.g., a top 10 music list demands 45 pairwise comparisons (Isaac & Schindler, 2014). In marketing research, where many competing products are available on the market, comparisons across dozens of items are not unusual (Chrzan & Peitz, 2019).

By contrast, best–worst scaling (BWS) is a survey method that sidesteps this set size problem by capitalizing on people’s natural capacity to identify extreme preferences (Louviere, Lings, Islam, Gudergan, & Flynn, 2013)^1^. Briefly, BWS methods involve individuals indicating their most favored (i.e., best) and least favored option (i.e., worst) over small subsets of options. Despite its relative simplicity, preferences constructed using BWS are consistent, and respondents benefit from the cognitive ease of indicating their preference by choosing between extremes (Marley & Louviere, 2005).

In this paper, we directly compare *ranking* and *BWS* across three experiments that asked participants about preferences between ten life values (e.g., rank the importance of benevolence, stimulation, etc., Schwartz, 1994). To preface the results, our primary finding is that BWS methods exhibit greater test–retest reliability compared to rankings, even within time frames as short as minutes. Additionally, we find that elicited preferences for *ranking* and *BWS* methods are largely consistent though BWS methods provide the additional resolution to discern the relative strength of people’s preferences where rankings can only provide ordinal information.

Our work draws upon a rich vein of research comparing rankings and BWS. For example, Lagerkvist (2013) compared consumer preferences for beef-package labeling with rankings and BWS and found the methods *did not* concur with one another. Lee, Soutar, and Louviere (2007) compared rankings and BWS but without testing the consistency of preferences as they used a between-subjects design. Similarly, Lee et al. (2019) used life values to demonstrate test–retest reliability of BWS but without an explicit comparison with rankings. Our study builds upon these findings to conduct a complete comparison of test–retest reliability of both scaling methods. Together, our results privilege BWS as an informative, and readily accessible alternative to ranking methods with greater test–retest reliability.

### Preference elicitation

**Fig. 1:**
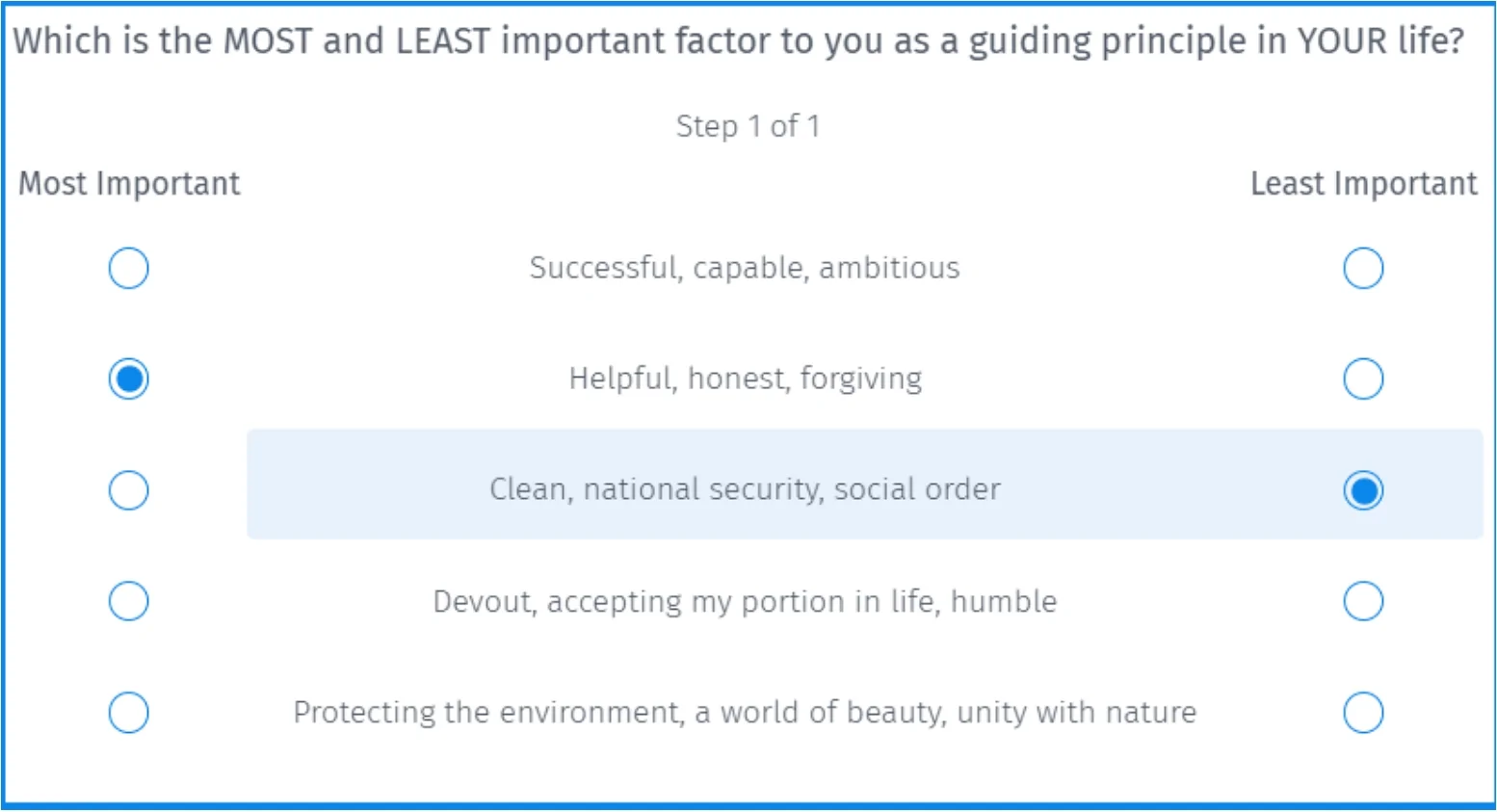
Example of a single best–worst scaling choice set involving five life values. In the BWS experiments, participants select the best (most important) and worst (least important) option from a set list. For this individual, the option “Helpful, honest, forgiving” was selected as the most important life value, and the option “Clean, national security, social order” was selected as the least important life value. The survey proceeds to a different set of options and over repeated choices of best and worst, preferences for all options are obtained. See Table 1 for full list of life value options used in our experiment where the materials were imported from Schwartz’s Values Theory used in Lee, Soutar, and Louviere (2008)

Best–worst scaling is a preference elicitation method that capitalizes on the cognitive ease of identifying preferences at the extremes (Louviere et al., 2015). In this paper, we consider case 1 ‘object’ BWS methods^2^ where individuals are presented with a set of items and asked to indicate their most *and* least preferred options within a set of alternatives. Figure 1 shows an example of a BWS choice where participants are presented with five options consisting of descriptions of life values. For this choice, individuals indicate from these five options, which is their ‘best-’ and ‘worst-’ preferred option before proceeding to the next choice comprised of a new set of options. Many BWS experiments adopt ‘case 1‘ designs to examine distinct options available in a market such as consumer food preferences (Llobell, Choisy, Chheang, & Jaeger, 2025)

The key advantage of BWS is that it reduces the cognitive demands for a decision-maker. At any single instance, an individual decides the best and worst on only a subset of the total options, such as in Fig. 1 displaying five of ten possible life value options. Preferences across all options are incrementally revealed over repeated choices with carefully balanced option subsets. These subsets are constrained such that, over the entire survey, each option appears an equal number of times and all options co-occur with each other with equal frequency (e.g., the “Successful, capable, and ambitious” option co-occurs with “Helpful, honest, forgiving” option a total of three times across different choice sets)^3^. This balanced design means that preferences across a large number of options can be broken into cognitively manageable piecemeal decisions and each best–worst choice is informative not only for the presented options but also places constraints on non-presented options from prior choices (Louviere et al., 2013).

**Table 1:** Dimension & item labels for all ten life values ordered by averaged task scores in Fig. 2. Participants were shown this table of definitions with both dimension & item labels at the outset of the experiment before completing the ranking or BWS choice task with only a *single* set of labels at any one time

	**Dimension labels**	**Item labels**
*1*	Benevolence	Helpful, honest, forgiving
*2*	Self-direction	Creativity, curious, freedom
*3*	Achievement	Successful, capable, ambitious
*4*	Security	Clean, national security, social order
*5*	Universalism	Protecting the environment, a world of beauty, unity with nature
*6*	Hedonism	Pleasure, enjoying life, self-indulgent
*7*	Stimulation	Daring, a varied life, an exciting life
*8*	Tradition	Devout, accepting portion in life, humble
*9*	Conformity	Politeness, obedient, honoring parents & elders
*10*	Power	Social power, authority, wealth

Best–worst choices also provide the resolution to quantify the strength of preferences between options. Because individuals make repeated choices with different options each time, BWS constructs preferences scores for each option from multiple comparison points. These added points provide information beyond rank ordering that allow for quantitative comparisons between options. This quantification is particularly useful where *qualitatively* distinct options may need to be weighed against one another. For example in health settings, Ejaz et al. (2014) asked oncology patients what information helped determine their choice of surgeon. While ‘surgeon training’ was centrally important to patient decisions, BWS methods provided the added resolution to identify that it was twice as important as ‘surgeon experience’ and over 3 times more important than the ‘hospital’s reputation’. Taken together, BWS provides rich data from relatively simple responses to understand the breadth of trade-offs in decision-maker preferences.

In this domain, ranking tasks are a natural comparison. Ranking is quick, and, for respondents, the goal is immediately apparent even if the exact individual ranks are not. These advantages make ranking inherently appealing as a simple, cognitively inexpensive way to elicit ordinal preferences from individuals. From a data perspective, rankings are computationally cheap to generate and a useful compression method in circumstances where only the top of the list is needed (Chun & Larrick, 2022).

Across either elicitation method, expressed preferences are subject to measurement error. This can arise due to idiosyncratic individual factors, such as ranking foods while satiated, using response styles that avoid the extremes of a rating scale (Grimmond, Brown, & Hawkins, 2025), or particulars of the elicitation method itself, such as a ranking task that prevents expressions of equivalent preferences between options. This added randomness to expressed preferences means our comparison of ranking to BWS methods cannot inherently privilege either as closer to the ‘true’ state of individual’s beliefs. Furthermore, like with music taste, there is often no ‘ground truth’ by which to compare subjective elicited preferences.

To remedy these complications, we incorporate three features into our experimental design.

First, to limit the possibility that changes reflect shifts in underlying preferences, our investigations focused on people’s life values. We chose this domain because values are personally relevant and preferences naturally vary across people (see Appendix for analysis). Our experiments utilized a previously validated set of 10 life values based on a condensed BWS version of Schwartz’ Value Theory (Lee et al., 2008; Schwartz, 1994). The main benefit for our investigation is that people’s life values are typically stable, and fundamental changes occur on timescales of years that dwarf our experimental demands of mere minutes (Rokeach, 1973; Schwartz, 1992). In addition, extensive work has delineated each life-value, providing a strong empirical basis for interpreting diversity of preferences across the set (Lee etal, 2019; Lindeman & Verkasalo, 2005). By comparison, preferences in applied decision environments, such as marketing research about consumer products, are often messier, subject to large contextual factors in time, location, and more-transient preferences for alternative options. Our intent is that by examining the relatively stable life-values domain, here, we provide an ideal testbed to understand the influence of test–retest reliability in applied settings.

Second, our experiments investigate whether elicited preferences persist when their descriptions change. Returning to our music analogy, consider that when describing the Beatles, one could define a broad genre (e.g., rock or pop) or their characteristic qualities (e.g., iconic counter-melodies, chord progressions, vocal harmonies). This choice of description highlights distinct features within a broader concept. Along a similar vein, our experiments describe the set of life values along broad *dimensions*, such as ‘benevolence’, as compared to example qualities henceforth called *items*, such as ‘helpful, honest, & forgiving’ (see Table 1). We anticipate that dimension- and item-descriptors will evoke different interpretations across individuals. Over and above these interpretations, however, we seek to understand whether rankings or BWS better preserves the elicited preferences across changes in description when both levels of description ostensibly target the same latent psychological concept.

Third, to address the absence of an underlying ground truth, we elicit preferences from individuals twice in all three experiments. From a random utility perspective, preference stability over time is one indicator of validity in the absence of ground truth. To control for changes over time, we first experimentally determine the test–retest reliability of a) rankings in Exp. 1a, b) BWS responses in Exp. 1b, before c) comparing elicited preferences across methods in Experiment 2.

## Methods

To highlight the consistency across experiments, we describe all three experiments together.

### Participants

Participants were recruited from the online platform Prolific Academic with the constraints that a) English was their first language, b) they had an approval rating greater than 80%, and c) participants did not participate in multiple experiments. Exp. 1a recruited 110 participants ($M_{age} = 41.7, SD = 12.9, N_{female} = 57$, $N_{male} = 52, N_{opt out} = 1$), Exp. 1b recruited 101 participants $M_{age} = 41.3, SD = 15.1,$ $N_{female} = 55, N_{male} = 44, N_{opt out} = 2$), and Exp. 2 recruited 161 participants ($M_{age} = 36.1, SD = 11.8$, $N_{female} = 66, N_{male} = 95$). From these experiments, we removed 9, 1, & 13 participants, respectively, where the experiment was only partially completed, where data were not saved, or where participants took longer than the maximum allowable time to complete the study (44 min).

Participants were paid at a rate commensurate with £9.00 per hour (Med$_{time} = 6.75$ min). In total, these criteria left respective *N*’s of Exp. 1a =101, Exp. 1b =100, and Exp. 2=148.

### Design

All experiments followed a two-task structure where preferences for ten life values were elicited twice. The experiments used a within-subjects (measurement occasion: first vs. second measurement) by between-subjects design (task labels: items vs. dimensions).

In Exp. 1a, preferences were elicited twice using the ranking task. In Exp. 1b, preferences were elicited twice using the BWS task. In Exp. 2, participants completed both ranking and BWS tasks, where the order of the tasks was randomized (i.e., ranking-first or BWS-first).

We manipulated the labels of the life values across both elicitation methods and measurement occasion. Values were labeled either as dimensions (e.g., hedonism) or example value-items (e.g., “Pleasure, enjoying life, self-indulgent”, see Table 1 for the full list of labels). These label sets were drawn from the Schwartz Value Survey (Schwartz, 1994), where previous work validated the items as representative examples of the ten life values (Schwartz, 1992; Spini, 2003).

We randomized the label set (item vs. dimension) between subjects. In Exp. 1a (ranking) and Exp. 1b (BWS), value labels were randomised such that $\frac{1}{3}$ participants completed both tasks with dimension-labels, $\frac{1}{3}$ with item-labels, and the remaining $\frac{1}{3}$ with label-changes across tasks; that is, we assume the order of completing the dimension- vs item-labels measure is inconsequential. In Exp. 2, we randomized labels across both task types and measurement occasions. That is, participants might see dimension labels *or* item labels at both measurement occasions, or they might see one of each label type across the two measurement occasions.

### Materials

All three experiments elicited value preferences using two methods: a ranking task and a BWS task. Both tasks were conducted through the online survey platform QuestionPro, and participants entered their responses using a mouse.

#### Ranking task

In the ranking task, participants were presented with a randomized list of ten values labeled as either dimensions or items (see Table 1). To order the list, participants were instructed to drag individual life values into a separate field that automatically ordered the list from most (1) to least (10) important. Once all items were transferred to the ordered list, participants could proceed to the next page.

**Fig. 2:**
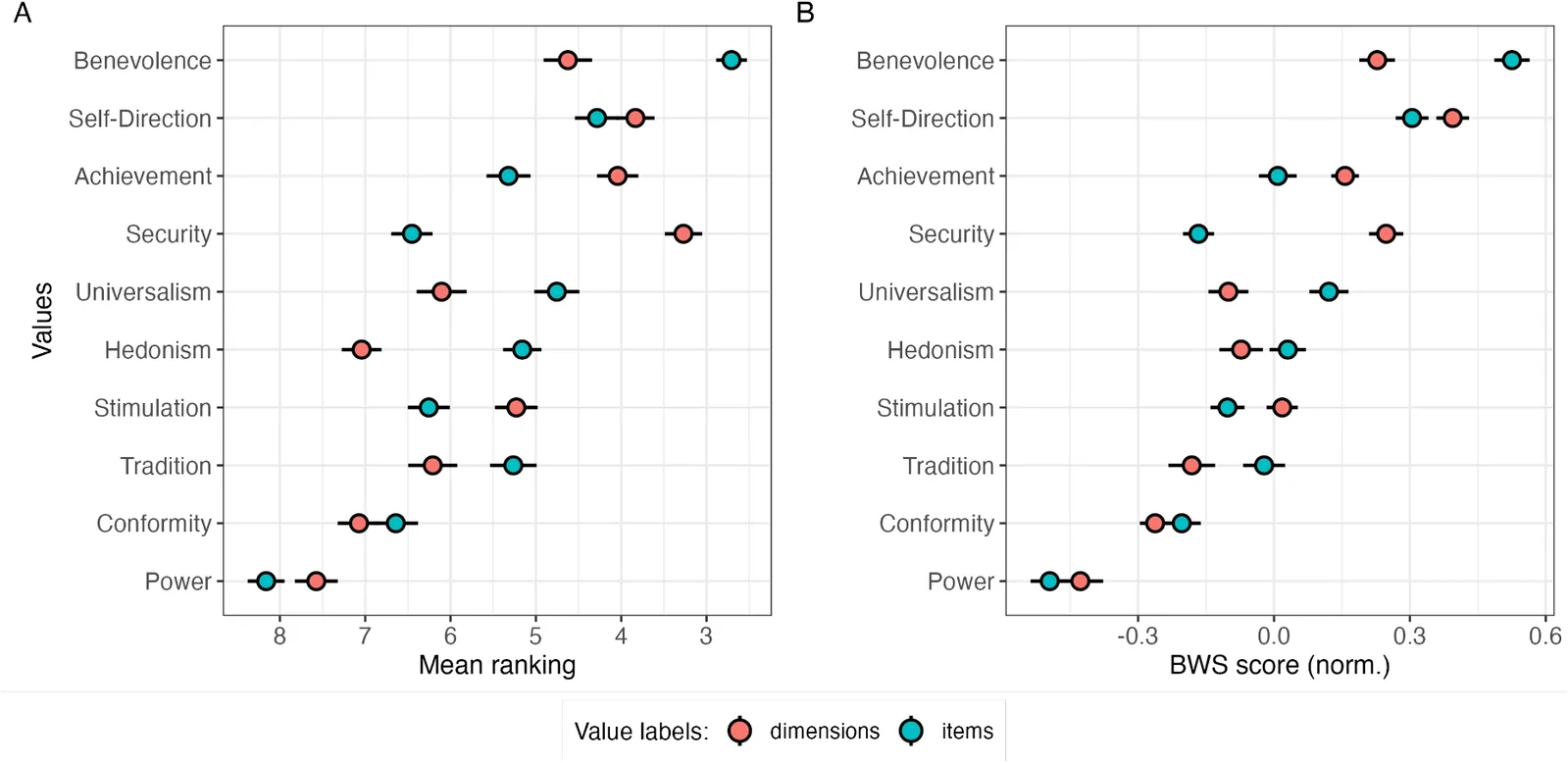
Overview of life value responses separated by task. *A* Mean ranks from Exp. 1a of each life value ordered from most important (1) to least important (10). *B* Mean BWS scores from Exp. 1b of each life value in the same order from most important (1) to least important (-1). Value label is shown in *color* and *error bars* represent the standard error of the mean

#### BWS task

We adopted a ‘case 1’ object-type BWS task used by Lee et al. (2008) to examine responses about life values, using an exact replication of their stimulus set structure (see their Table 2). This stimulus set consisted of 11 subsets of Schwartz’s life values, such as ‘hedonism’ and ‘power’, and asked participants to indicate the most- and least important life values within each subset (for example, see Fig. 1).

We generated the specific choice sets using a balanced pairwise design to balance the number of comparisons and presentations of each life value option. Specifically, each subset consisted of lists of either five or six options (life values) and, in total across the subsets, each life value was presented six times. The complete list of life values is presented in Table 1. Subsets were balanced such that pairs of values co-appeared on only three occasions. For example, ‘hedonism’ and ‘power’ appeared in the same subset on three occasions where the remaining values in each subset differed on each occasion. This randomization within sets ensures that each best–worst selection is weighed evenly against all remaining items shown in other subsets.

Participants were instructed to select the most- and least-important value within each subset before progressing to the next subset of values. Values were presented as a list with a ‘most’ indicator on the left of the list and a ‘least’ indicator on the right. There was no order restriction as to which preference was selected first. The 11 subsets were presented in a randomized order, and the individual options within each set were not randomized.

### Data processing & outcomes

To compare each method on common ground, our analyses elicit a list of preference orderings from both the ranking and BWS tasks. For the ranking task, we preserved the raw ranks ranging from 1st to 10th. For comparison, the BWS task offers a range of statistics from which to generate an ordered list^4^ and, so for ease of presentation, we analyzed responses using normalized best–worst scores (Lipovetsky & Conklin, 2014; Louviere et al., 2015; Marley, Islam, & Hawkins, 2016). Normalized BWS scores can be converted to an ordered list comparable to ranks, but they can also be analyzed to have the added resolution of relative distances between ranks at the participant-level. For our main analyses comparing the two elicitation methods, we primarily focus on the rank order of these lists.

From Eq. 1, normalized best–worst scores are generated by a count of ‘best’ and ‘worst’ selections for each life value, where *i* indicates a single life-value from our set of 10. Normalization centers the scores around a 0 value where positive values indicate more selections of ‘best’ for a given life value, and negative values indicate more selections of ‘worst’ for a life value. B-W values are normalized over the number of item presentations across the experiment, where ${N_i(appearances)} = 6$ in our experiments. Normalization bounds the scores between -1 and 1 where values close to 0 indicate non-preference. A BWS score of 0 is possible if an option is never chosen as either the best nor the worst across all responses, or if an option is chosen as best the same number of times it is chosen as worst. For reference, in Experiment 1b where the BWS task was repeated twice, an average of 2.28 life values (SD=0.90) out of ten possible life values were never chosen as best nor worst.

The B–W scores for each life value are then arranged from highest to lowest to form a rank-ordered list that is compared against the ranking task.

1 $$\begin{aligned} \frac{N_i(\text {\textit{most} selections}) - N_i(\text {\textit{least} selections})}{N_i(appearances)} \end{aligned}$$

**Fig. 3:**
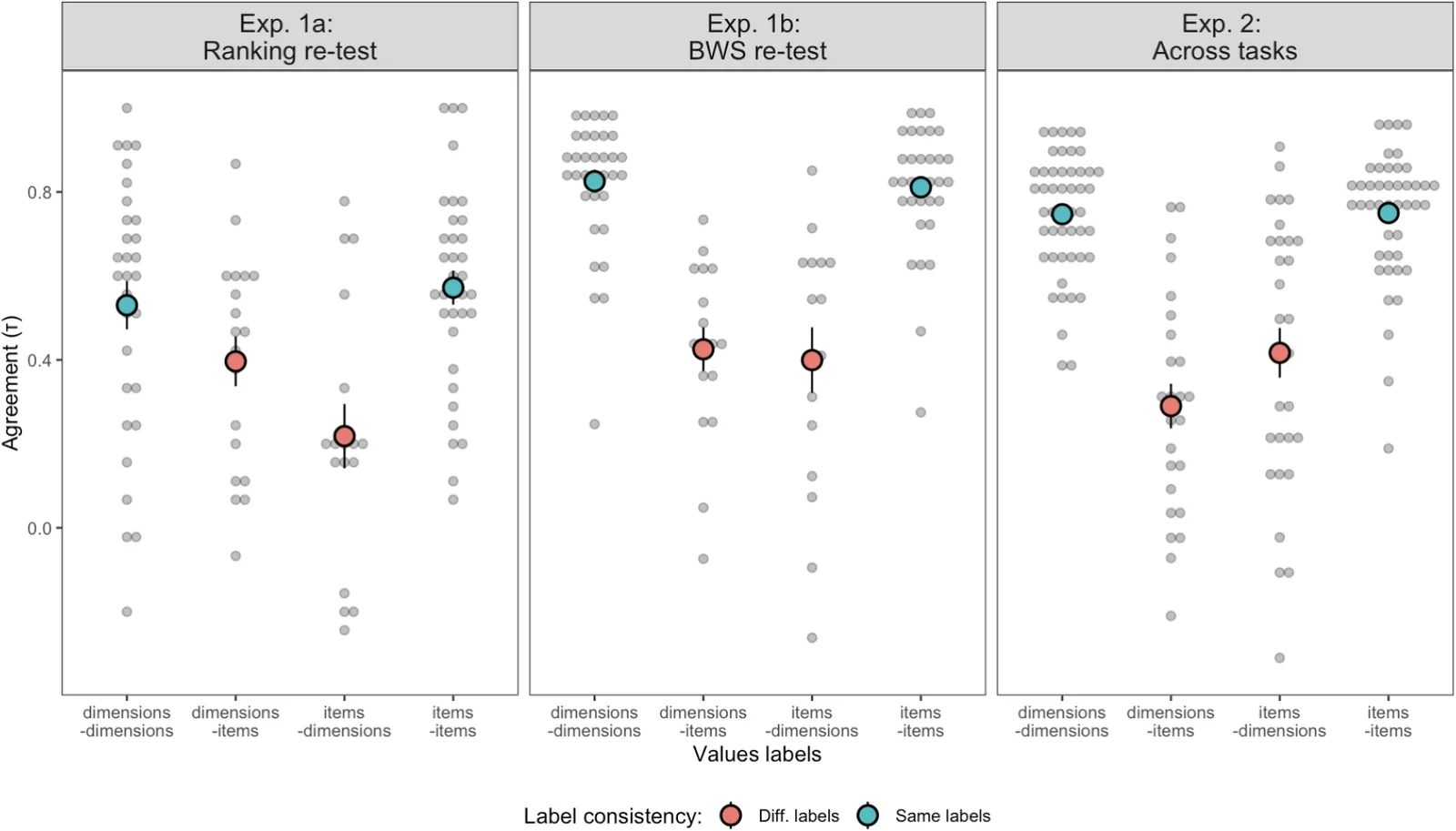
Agreement across measurement occasions as a function of life value labels (*x*-axis) and experiment (panels). * Gray dots* show individual-level participant data and larger *colored dots* show means and standard error. Color indicates life value label consistency. *Blue colored dots* indicate conditions where life value labels were the same on both measurement occasions, and *red color dots* indicate when labels differed. Exact ordering of labels is shown on the *x*-axis. See Table 1 for complete list of life-value labels

### Procedure

The three experiments each started the same. Participants began the online experiment by clicking a link to the experiment web page. At first, they were told the experiment was investigating life values and shown a table of values with both *dimension* and *item* labels, similar to Table 1. Participants were then randomly allocated into label conditions where value labels remained identical across both measurements (e.g., item-item or dimension-dimension) or value labels alternated (item-dimension or dimension-item).

Participants completed their first response task, which was either a ranking task in Exp. 1a, a BWS task in Exp. 1b, or a random selection between the two tasks in Exp. 2. Once complete, participants proceeded to the second task under the same constraints. For Exp. 1a and 1b, this was the same task they completed in their first response task, though dimension vs item labels may change. In Exp. 2, the second task was the task *not* completed first (i.e., if the ranking task was completed first then BWS was second, and if BWS was first then ranking was second). Participants were reimbursed upon completion.

## Results

As an initial overview of the data, we first present an overall summary of participant preferences for the ten Schwartz life values. Figure 2 panel A presents mean life value ranks from Exp. 1a and panel B presents mean BWS scores from Exp. 1b. Almost entirely, ranks and BWS scores were in agreement. For both tasks, the most important value was *benevolence* ($M_{rank} = 3.62, SE = 0.18$, $M_{BWS} = 0.375, SE = 0.03$) and the least important value was *power* ($M_{rank} = 7.88, SE = 0.17$, $M_{BWS} = -0.46, SE = 0.03$). Notably, the top five most important life values were ordered identically between the ranking and BWS tasks, as were the two least important life values. Only a single pair of values, *tradition* & *hedonism*, ranked 6th or 8th, traded ordinal positions between the preference elicitation methods.

As a reminder, across all three experiments, participants completed two preference elicitation tasks. In the next section, we present the agreement between both measurement occasions using Kendall’s Tau (τ). Kendall’s Tau is a non-parametric statistic of rank correlation for ordinal data that provides a metric of similarity for lists of life-values generated by BWS as compared to ranking (Kendall, 1945). Tau values are bounded between 1 & -1, where τ values of 1 indicate complete agreement across both lists, and τ values of -1 indicate exact reversals. For clarity, we use the $\tau _{b}$ variant of Kendall’s Tau that explicitly accounts for ties between lists. Using the overview data from Fig. 2 as an illustration, the order of mean ranks strongly agreed with mean BWS scores (items: τ=0.96, $BF_{10} = 221.4$, dimensions: τ=0.82, $BF_{10} = 43.0$, Fig. 2)^5^.

### Experiment 1a (ranking) & 1b (BWS)

We first examine the preferences across repeated-measurement occasions within the same task (Exp. 1a & 1b) and then across elicitation tasks (Exp. 2), shown in Fig. 3. Note that in the following sections, agreement between measurements is indexed by Kendall’s Tau (τ), which is shown at the individual participant level within the figure and reported in the aggregate in the text.

Beginning with Exp. 1a & 1b, the agreement across first and second measurements was higher, on average, for the BWS task compared to ranking task (i.e., the average of individual participant Kendall’s Tau values shown in the left-most vs. center panel Fig. 3, $M_{rank} = 0.47$ vs. $M_{BWS} = 0.69$, *SE’s*=0.03, BF>1000). This advantage for BWS tasks likely emerges from the relative ease of selecting preferences at the extremes. In line with this interpretation, the most common *rank* to change across measurement occasions in Exp. 1a was 6, one of the middle ranks (81% of rank 6 responses changed), and the consistency of ranked values across measurement occasions only increased for each rank-step toward either extreme. That is, by comparison, rank 1 changed for only 54% of responses, rank 10 responses changed for 62%, and other rank-changes monotonically increased with each rank-step before peaking at rank 6.

**Fig. 4:**
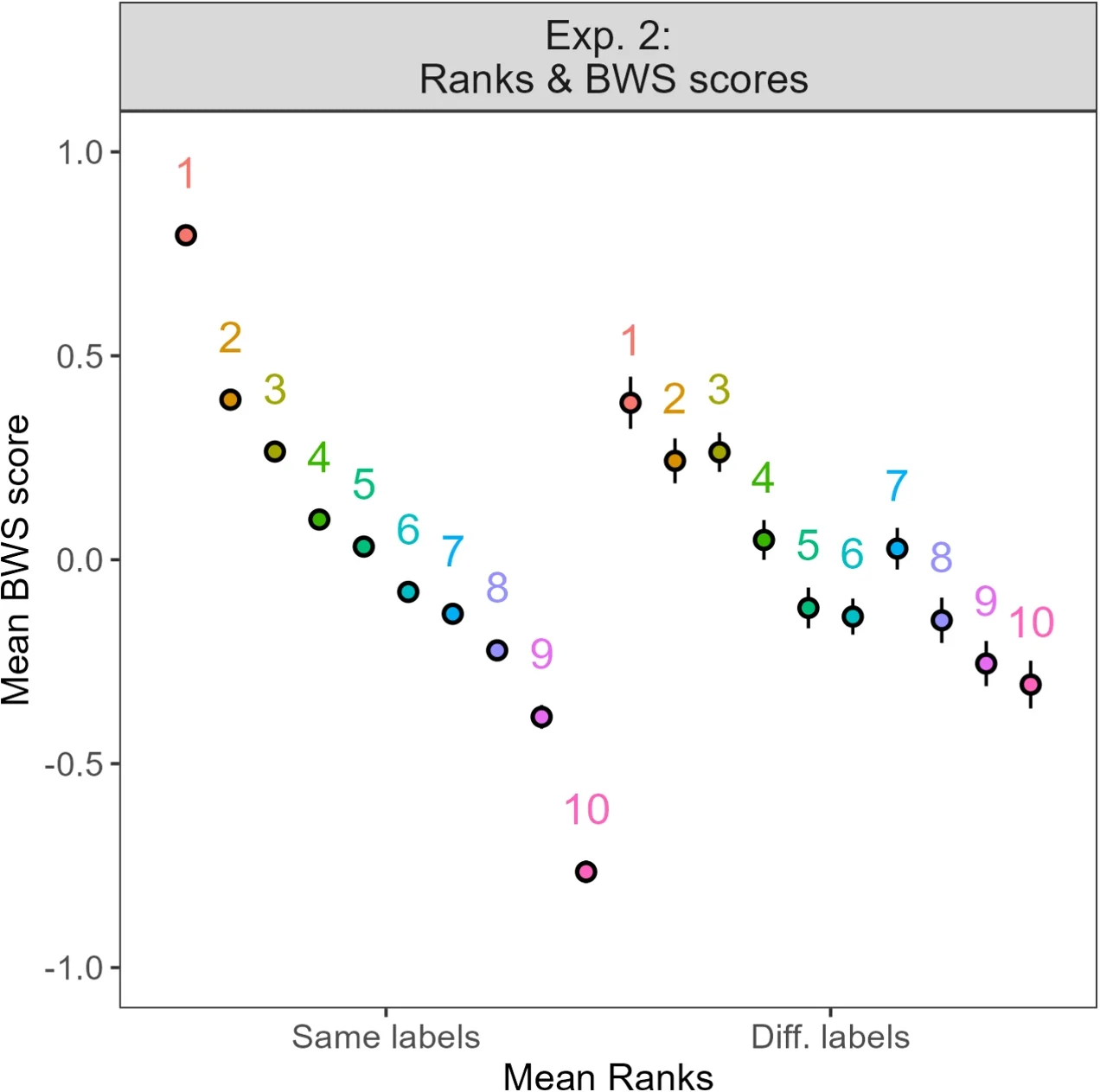
Experiment 2 aggregate-level data showing mean BWS scores (*y*-axis) as a function of ranks (*x*-axis). For each rank position, the mean BWS score across individuals is shown in the colored points. Error bars represent standard error of the mean, and text indicates corresponding rank positions. Between-subject label conditions are separated on the *x*-axis. As a reminder, *different labels* indicate dimension-item labels switched with ranking and BWS tasks whereas *same labels* indicate consistent labeling across tasks

Across both Exp. 1a & 1b, agreement was stronger with consistent life-value labels than inconsistent labels (blue vs. red points, Fig. 3, $M_{1a} = 0.55$ vs. 0.31, $M_{1b} = 0.82$ vs. 0.41, BF>1000). That is, changing the label of the life values between measurement occasions decreased the agreement between surveys (e.g., benevolence ↔ helpful, honest, forgiving).

Notionally, agreement should differ if the new label evokes qualitatively different definitions of a particular life value. This divergence in people’s interpretations of item and dimension labels appears in the aggregate preference, shown in Fig. 2. Despite the divergence, however, one interesting consistency is that across both ranking and BWS tasks, the relative preference for an item or dimension label for any *individual life value* was the same (red vs. blue points across panels in Fig. 2). From a construct-validity perspective, this consistency is reassuring, as it indicates that despite changing elicitation methods, we indexed the same underlying preferences for each label set, at least in the aggregate. We return to this point in the General Discussion.

One point worth noting is that the BWS task allows for ties in preference where ranks do not. These ties indicate equivalent (non-)preferences between life values and so for ten life values, there may be fewer than ten bins of BWS scores. In Exp. 1b, where individuals completed the BWS task twice, the mean number of BWS-score bins was similar across the first measurement occasion and the second measurement (M=7.06 vs. 6.89, $BF_{01} = 2.82$). Notably, both occasions exhibited fewer than ten bins, indicating the presence of ties in preferences where ranks in Exp. 1A may have forced discrimination.

### Experiment 2 (crossed-tasks)

In Experiment 2, we directly compared preference-elicitation methods by having participants complete both BWS and ranking tasks. Overall, preferences elicited by BWS methods largely agreed with preferences elicited by ranking (right-most panel, Fig. 3, $M_{\tau } = 0.60, SE = 0.02$). However, similar to Exp. 1a and 1b, consistent labeling led to greater agreement across ranking and BWS methods (blue vs. red points, $M_{\tau } = 0.75$ vs. 0.36, BF=9.19). To better understand where rankings and BWS specifically diverged, we now turn to examine raw BWS scores from Experiment 2.

Figure 4 shows the mean BWS scores for each rank position as a function of label consistency. Although both measures largely agree on ordinal terms, the relative distances between ranks may guide where disagreement is likely to emerge. For conditions with consistent labeling, one immediate observation is that BWS scores for the first and last rank are more distant from their neighboring ranks compared to any other rank positions ($\Delta _{1-2} = 0.40, \Delta _{9-10} = 0.38$ vs. $M_{\Delta } = 0.11, SE = 0.01$). This discrepancy suggests that preferences for first and last ranked values are generally stable. By comparison, average BWS scores for middle ranks, such as the fourth, fifth, and sixth ranks, are more tightly clustered, suggesting that preferences for options in these rank positions are less discernible from one another. The conflation of similar BWS scores is particularly exaggerated for conditions where the labels switched between tasks (right-most *x*-axis points, Fig. 4). Broadly, the distribution of ranks is more centrally concentrated and within each rank position, each mean BWS score is more variable compared to conditions with the same labels. Considered together, mean BWS scores across rank positions provide a tentative guide as to where disagreement between ranks and BWS arises.

## General discussion

In this paper, we contrasted two popular methods of preference elicitation. We compared rankings, which extract ordinal preferences, to BWS methods that additionally provide relative information about the strength of those preferences. Across three experiments, we examined the agreement between elicited preferences over time in Exp. 1a and 1b, across elicitation methods in Exp. 2, and over changes in item labels. Together, we draw two main conclusions.

First, preferences elicited from BWS and ranking agree with one another. Despite the differences in response methods and task demands on individuals, we find that elicited preferences were largely consistent, indicating that both methods captured a common underlying representation of individual’s preferences about their life values. For survey-designers, our findings provide an empirical validation to support using BWS in contexts where ranking methods are currently employed. The primary benefit is that not only are ordinal preferences between methods preserved but BWS also provides valuable relative information between preferences and detects ties in preference where rankings cannot.

Second, BWS has greater test–retest reliability compared to rankings. A core methodological consideration is whether one’s measurements are stable over time. From Experiment 1a, we find that while acquiring a set of rankings is relatively inexpensive in time costs, not all ranks are valued equally. Specifically, test–retest reliability across sets of rankings suffers when middle ranks are interchanged, since they are generally less discernible than preferences at the extremes. BWS capitalizes on this exact psychological feature. We found that the cognitive ease of identifying the best and worst options in BWS produces higher test–retest reliability across measurement occasions particularly for middle rank preferences that are more prone to interchanging across occasions.

Taken together, our results privilege BWS over rankings as it provides a richer characterization of preferences alongside the reassurances of test–retest reliability.

### Different labels, same interpretations?

Our work leaned on the conceptual similarities between items and dimensions. Across experiments, we labeled each life value using either a dimension label (e.g., benevolence) or an item label (e.g., helpful, honest, forgiving). These labels were drawn from labels sets within Schwartz’ Value Survey (Schwartz, 1994) where previous work conducted across 21 countries had validated the set of items as representative examples of the ten life values (Schwartz, 1992; Spini, 2003).

When thinking about life values, however, context provides many cues to guide interpretation. For instance, the personal importance of the life value ‘power’ is likely to differ if one individual defines power as social influence while another individual defines power as a proxy for wealth. Alongside individual differences, however, one important consideration is whether the elicitation method itself led to divergent definitions. If one method inherently guides individuals toward a particular definition, then agreement between methods would be systematically compromised by the item set rather than the method. We addressed this possibility by incorporating two sets of item labels and comparing the preferences extracted from each.

Across experiments, item and dimension label sets led to different interpretations of the life values. Most apparent is that, in all three experiments, agreement was lower when label sets alternated. Notably, this disparity held for twice-attempted ranking tasks in Exp. 1a and BWS in Exp. 1b, suggesting that different interpretations were present independent of the elicitation method.

Despite the differences *between* the label sets, one interesting consistency is that their definitions appear to have been shared *across* elicitation methods. This convergence is evident in the relative preference data. In Fig. 2, the *order of preference* for either item or dimension labels is consistent across ranks and BWS scores for every life value. For example, ‘benevolence’ was more preferred when labeled as items ‘helpful, honest, forgiving’ compared to as a dimension *in both tasks*. Similarly, ‘security’ was more preferred when labeled as a dimension rather than as items ‘clean, national security, social order’. Interestingly, not only is this ordinal preference consistent across elicitation methods, but the relative magnitude in preference between item- and dimension- labels is also consistent. That is, in the aggregate, the *relative degree* to which our participants preferred ‘benevolence’ when labeled as items ‘helpful, honest, forgiving’ compared to as a dimension is similar in both rankings and BWS scores, and this relative consistency is present for all life values except for ‘hedonism’.

Considered together, the primary benefit of our labeling manipulation is that it provided an additional testbed for the generalizability of our findings. Despite the very different response methods and measurement scales, participants for both rankings and BWS methods converged on common ground for each set of labels^6^. This consistency means that within the same choice context of life values, our experiments provided two comparisons between the elicitation methods, broadening the foundations of our conclusions beyond a single stimulus set. More generally, two sets of labels also strengthen our methodological comparison through a constructed-preferences lens. If preferences are indeed *constructed* at the moment of measurement, a single stimulus set leaves open the possibility that one particular elicitation method is privileged by the operationalization of the domain. Instead, our results suggest that the task differences between ranking and BWS did not by alter our participants’ preference structures and so differences in test–retest reliability are attributable to the elicitation methods themselves.

### Considering set size

Compared to rankings, a core benefit of BWS is that preferences across larger sets are as easily obtained as preferences for smaller sets. For only ten life values, our experiments found that rankings in the middle order were more likely to change across measurement occasions. Hypothetically, one might expect that increasing the set size would only exaggerate the paucity of information from middle-ranked values while adding greater cognitive effort of comparing each item against a larger set of alternatives. When one considers the large number of possible influences on surgeon choice (*N* = 16 in Ejaz et al., 2014) or the available response options in personality surveys (*N* = 57 in Schwartz, 1994), it becomes clear how ranking each option relative to a full set can be unwieldy.

BWS sidesteps this set-size problem because of its iterative, piecemeal design. The main mechanism that permits this is that for any set size, BWS methods require only two choices from a subset of all items. For example, our experiments presented subsets of five or six life values, from which only the best and worst are chosen. This feature means larger set sizes only increase the number of choice ‘rounds’ an individual completes while maintaining the same cognitive load demand across each round.

Naturally, this advantage is not free. Increasing the number of rounds means that for the respondent, BWS surveys can take longer to complete as compared to equivalent set-size ranking surveys, as was borne out in our completion time data (Exp. 1a vs. 1b, $$M_{rank} = 4.60$$ vs. $$ M_{BWS} = 8.60$$ min). However, our findings suggest that the added reliability afforded by the time cost is worthwhile.

More broadly, our work dovetails with the long-standing tradition of examining preferences in the absence of ground truth. Like with music taste or choosing between medical treatment options, understanding the subjectivity is the objective. One notable and recent exception is that of Gronau, Bennett, Brown, Hawkins, and Eidels (2023) whereby classic likert elicitation methods and choice tasks are compared in settings where a ‘truth’ was engineered, albeit modifying the standard preference elicitation use case. Akin to their efforts, the goal of this work was to evaluate two popular elicitation methods by standards of reliability, preserving their core task qualities – subjective warts and all. By these standards, we arrive at the conclusion that over time, the BWS method is more reliable than ranking.

## Figures and Tables

**Fig. 5:**
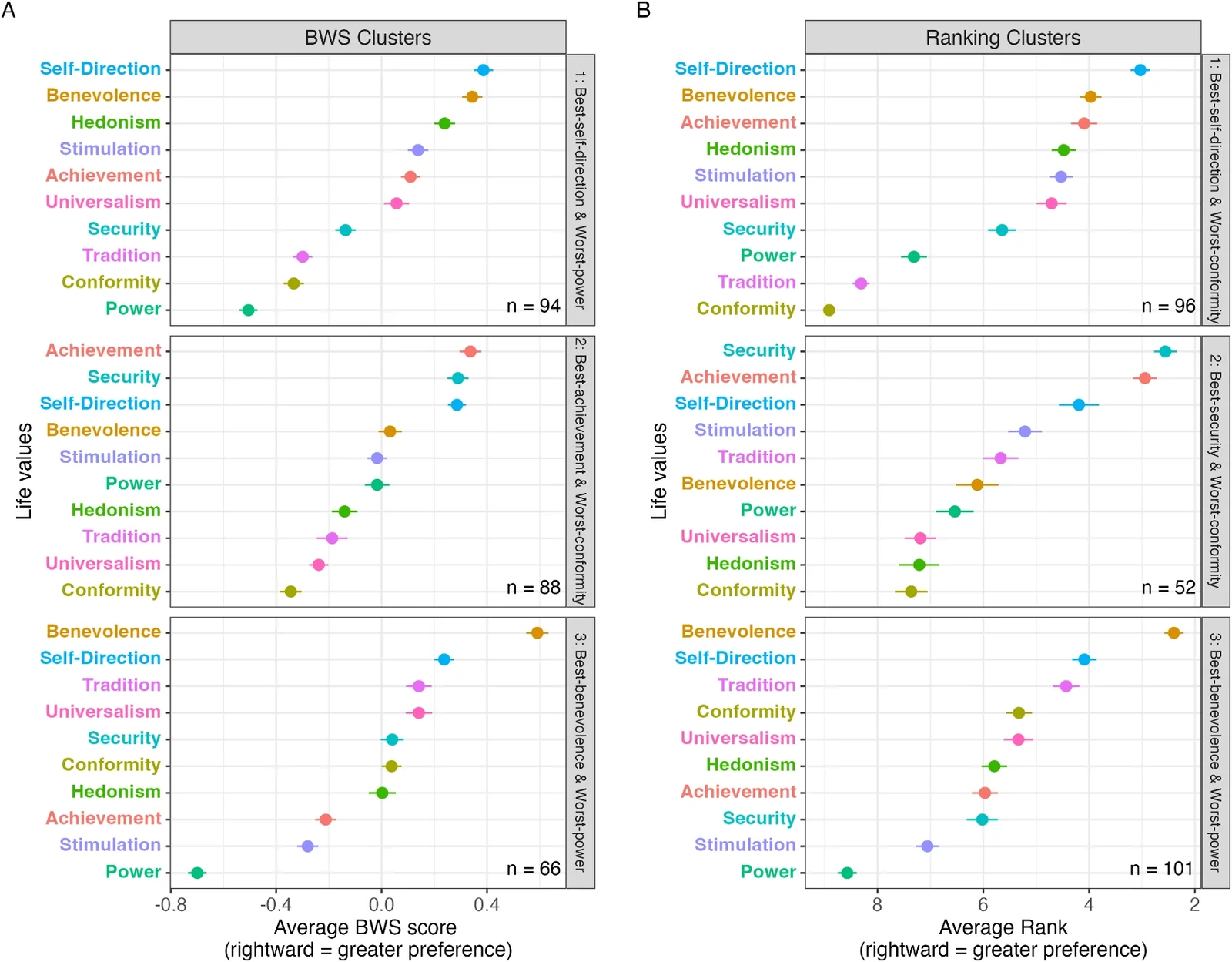
Preferences for life values by sub-groupings of participants. Horizontally, panel *A* shows three BWS-generated subgroups and panel *B* shows three ranking-generated subgroups. Vertically, each cell shows a distinct sub-grouping ordered by their most-preferred and least-preferred life values. *Point values* and *error bars* represent means and standard errors, where the *colors* of each point correspond to the colored life value *y*-axis labels, where colors are consistent across all six cells. Corresponding *N*’s for each sub-grouping is printed within the cells

## Data Availability

Survey materials were collected on the QuestionPro platform and can be shared on request. Raw and preprocessed data are available on OSF. https://osf.io/ct8ae/

## Appendix

### Diversity of preferences

Here, we showcase the diversity of preferences across individuals in our experiments. The broad finding is that distinct preference clusters were disguised in the aggregate. Importantly, and converging with our main analyses, we observe that both elicitation methods converged on identifying clusters with similar preferences, and we show these in Fig. 5.

Sub-groupings were generated through clustering analysis of preference responses. Separately for each task, ranking and BWS score profiles were compared across participants using Kendall’s Tau correlations, converted to a distance metric (1-τ), and clustered with Ward’s hierarchical clustering to assign respondents to groups with similar preference structures. For the purposes of illustrating diversity of preferences, we include data for each individual’s first attempt at each task from Experiment 1a, 1b, & 2 without their corresponding second attempts. That is, ranking data consisted of the first attempt at Exp. 1A and the ranking portion of Exp. 2, and BWS data, consisted of the first attempt from Exp. 1B and the BWS portion of Exp 2.

In Fig. 5, panel A shows sub-groupings clustered by mean BWS scores and panel B shows sub-groupings from mean rank clusterings. For ease, we label the sub-groupings based on their ‘best & worst’ preferred life-value. For example, BWS cluster 1 in the upper-left-most panel shows similar life-values ordering to Rank cluster 1 in the upper right-most panel. Both methods identified a cluster of individuals that valued “Self-Direction” and “Benevolence” the most, and did not value “Power”, “Conformity” and “Tradition” albeit in different orderings in the tails.

For illustration, it’s helpful to compare this first cluster to the third cluster in the bottom of panels A & B. The third cluster is similar to the first in that “Benevolence” and “Self-Direction” are the most preferred life values, though by contrast, the third-most preferred value is “Tradition” which has low preference in the first cluster. Similarly, the third cluster shows a common low preference for “Power” but also “Stimulation” which is ranked relatively highly in the first cluster. Overall, there is a broad diversity of preferences within our sample and this diversity was detected by both measurement tasks (panels A vs. B).

## Footnotes

1 For the reader interested in using BWS themselves, we refer to Louviere et al. (2013) for an applied demonstration of BWS and Louviere, Flynn, and Marley (2015) for a comprehensive guide.

2 For any set of items, the three BWS cases represent different levels of specification that an experimenter wishes to compare. The case 1 ‘object’ case is where each item is a distinct object from the other options (e.g., oranges vs. apples). The case 2 ‘profile’ case is where participants are presented with a single item that is compared in terms of its attributes (e.g., an apple with two attributes of varying sweetness and color) and individuals select the best and worst *attributes* within the profile. Case 3 combines both ‘object’ and ‘profile’ cases where individuals select a preferred profile amongst a set of profiles (Apple A vs. Apple B vs. Orange A each with attributes sweetness & color). Each case is not mutually exclusive as such but chosen to best suit the question of interest. For a collection of applied medical examples, Cheung et al. (2016) identified 62 BWS studies used in healthcare with examples of each BWS case type which has subsequently been updated in Hollin, Paskett, Schuster, Crossnohere, and Bridges (2022) ’s recent systematic review.

3 At the time of publication, the ‘support.BWS‘ R package helpfully provides a general implementation for designing stimulus sets, and analyzing the BWS data (Aizaki & Fogarty, 2023). Key experimenter-controlled decisions such as total number of item presentations, number of alternative options, and number of co-occurrences with other options can be set by the user depending on their experiment parameters. We note that for reliability, we borrowed the stimulus set from Lee, Soutar, and Louviere (2008), which satisfies a balanced pairwise design, and refer readers to balanced incomplete block designs (BIBDs), the gold-standard framework for constructing such balanced choice sets.

4 For a comprehensive review, see Cheung et al. (2019) who compared five statistical BWS metrics, e.g., best–worst score count analysis used in our analyses, multinomial logit models, & latent class analyses, and found that on a common rank comparison, the metrics agreed with one another.

5 For ease of interpretation, we report all Bayes factors for and against the null as values >1 and follow Jeffreys (1998) ’s scale where Bayes factors > 10 indicate strong evidence.

6 We note this convergence is in the aggregate and is not to imply that every individual preferred the same life values. In the Appendix, we point to profiles of individuals that appeared within our data and show a broad diversity of preferences across individuals.

## Code Availability

Analysis & data preparation code are available at the above OSF repository.
